# Artificial Intelligence for Clinical Decision Support in Rural Spine Care: A Narrative Review

**DOI:** 10.3390/healthcare14182935

**Published:** 2026-09-10

**Authors:** Aviraj Soin, Charles A. Odonkor, Massab Bashir, Jose R. Rodriguez

**Affiliations:** 1 The Ohio Pain Clinic, Centerville, OH 45459, USA; drsoin@gmail.com (A.S.); massab.khaira@gmail.com (M.B.); 2 Department of Orthopedics and Rehabilitation, Division of Physiatry, Interventional Pain Medicine, Yale School of Medicine, 47 College Street, New Haven, CT 06510, USA; 3 Geisel School of Medicine at Dartmouth, Dartmouth College, Lebanon, NH 03756, USA; jr.rod22@gmail.com

**Keywords:** artificial intelligence, machine learning, deep learning, clinical decision support, rural health, spine care, spinal pain disorder, low back pain

## Abstract

**Background/Objectives**: Artificial intelligence (AI) is increasingly applied across spinal pain care, by improving diagnostic accuracy, optimizing clinical workflow, and advancing translational research. However, AI’s role and its integration into rural spine care face challenges. Therefore, the current narrative review synthesizes the role of AI in rural spine care, spanning diagnosis, clinical decision support, imaging, natural-language processing, and remote monitoring. **Methods**: We searched the peer-reviewed literature on AI in spinal pain care across six databases from inception to June 2026. **Results**: In the United States, AI models have shown promise in strengthening clinical decision support that assists clinicians in identifying spinal pain disorders and providing evidence-based treatment recommendations; however, most were developed and validated in urban healthcare settings. Additionally, evidence on external validation, dataset representativeness, robustness to incomplete or low-quality data, interoperability, prospective clinical utility, and implementation feasibility in rural spine care remains limited. Therefore, we propose a translational framework in which de-identified data from rural and urban healthcare settings are aggregated, harmonized, and used to develop a multimodal AI model. AI models further require rigorous technical and external validation, prospective validation in rural settings, explainability, and continuous post-implementation monitoring. Despite its benefits, key limitations, including data scarcity and algorithmic bias, are also highlighted. **Conclusions**: AI has emerged as a promising tool for strengthening clinical decision support in spinal pain disorders in rural settings. The most realistic role of AI in rural settings is not to replace specialist expertise, but to act as one component of a multidisciplinary care team.

## 1. Introduction

Globally, spinal disorders such as low back pain (LBP), scoliosis, and spinal stenosis are crucial health problems that impose a significant burden on both patients and healthcare systems [1]. Among spinal disorders, LBP is the most prevalent spinal condition, with global prevalence rising to 452.8 million cases during 2021, which is 52.66% increase since 1990, and is the primary cause of disability [2]. Additionally, LBP causes a socioeconomic burden globally, with annual expenditures in the United States (US) alone exceeding $40 billion, $2000/patient/year, and still continuing to rise with increasing rates of imaging, high rates of surgeries or revision surgeries [3]. In addition, spinal cord injuries (SCIs), are sudden and debilitating events that cause long-term motor, sensory, and autonomic dysfunction, which disrupt community participation and quality of life (QoL). Therefore, comprehensive healthcare facilities can have a significant impact on promotion of long-term health of patients with these chronic conditions [4]. Although access to timely diagnosis and appropriate management is essential to reduce disability and prevent avoidable complications, the availability of specialized spine care remains unevenly distributed, and this is particularly evident in rural settings. Individuals with spinal disorders living in rural areas may experience multiple barriers in accessing healthcare facilities as compared to those in urban areas, such as timely access to primary care and specialized healthcare services, shortages of advanced diagnostic and treatment equipment, and transportation challenges [5]. Rural populations also face limited health-promoting activities, therapies, and mobility services, alongside financial barriers such as lower incomes and substandard health insurance [5,6]. Additionally, clinical data may be incomplete or inconsistently documented, electronic health systems may be heterogeneous, and diagnostic imaging may be acquired with equipment of variable technical capabilities [7]. Ultimately, these barriers may result in delayed diagnosis, increase disease severity, and lead to treatment of preventable conditions. Mitigating barriers for the population living in rural areas with spinal disorders would result in important improvements in treatment outcomes [4]. These challenges have forced researchers/clinicians into the development of innovative models of care that can extend the expertise of pain specialists beyond urban centers. Therefore, applying artificial intelligence (AI) models can improve diagnostic accuracy and reduce the need for complex spine surgeries for rural patients [8].

Artificial intelligence (AI) enables computers to perform tasks that typically require human intelligence, such as perception, pattern recognition, and decision-making [9]. Over the past decade, AI has transformed from a topic of theoretical interest into a practical clinical tool [10]. AI-based image analysis may assist with the detection, prioritization, and characterization of spine abnormalities when specialist interpretation is not immediately available [11]. The adoption of AI in healthcare has the ability to process large-scale clinical data through sophisticated algorithms and can match or exceed physician performance on specific, narrowly defined tasks [10]. Such systems may support risk stratification, diagnostic assessment, and triage and referral decisions by integrating clinical, imaging, and other essential patient-associated information [12]. AI may also support acute and chronic pain management, for example, by helping to predict opioid-related risk and personalize analgesic strategies [13]. Recent studies have extracted complex anatomical data from general radiography (X-rays), computed tomography (CT), and magnetic resonance imaging (MRI) to develop AI algorithms addressing anatomical, tissue-specific pathology associated with cervical, thoracic, and lumbar spine disorders. Furthermore, computer-vision algorithms analyze these images and identify predictive associations between patient outcomes and specific variations in the disc, bone, soft tissue, and spinal alignment [14,15]. These algorithms may also help predict length of postoperative stay and flag potential complications, readmissions, and alternative treatment options [16,17]. By extracting and synthesizing relevant information from clinical documentation, AI models could extend clinical expertise, improve the consistency of decision-making process, and support more efficient use of limited healthcare resources.

In recent years, the role of AI in spinal care has rapidly expanded, revealing innovative ways to optimize complex procedures and enhance patient care; its greatest value may lie in operating as an adaptive decision-support partner rather than an autonomous decision-maker. In spine surgery, where precision and planning are paramount, AI can support data-driven surgical planning. However, the potential relevance of AI models to rural spine care should not be equated with readiness for implementation. Most available AI models have been developed and evaluated using data from urban healthcare settings, their performance may be suboptimal for unique populations such as rural or high-altitude residents due to differences in patient demographics, disease presentation/severity, diagnostic/imaging equipment protocols, or data [18]. Rural deployment may also face challenges related to interoperability with heterogeneous health information systems, algorithmic bias, limited technical support, low-quality data, and the need for appropriate human oversight [19]. Therefore, this review summarizes the application of AI in rural spine care. We highlight the current evidence, challenges, and future opportunities of AI in rural spine care. The narrative synthesis was also guided by the following key research question: What evidence exist regarding the diagnostic performance, clinical validity, and generalizability of AI models for spine disorders in rural healthcare settings? By critically synthesizing the available evidence, this review moves beyond describing potential AI applications and proposes a translational framework to guide future research and validation.

## 2. Materials and Methods

Relevant literature was searched across electronic databases including PubMed, Web of Science, Scopus, Cochrane Library, ScienceDirect, and Google Scholar from inception to June 2026. Search terms included combinations such as “Artificial intelligence”, “Machine learning”, “Deep learning”, “Large language models”, “Spine care”, “Spinal cord injury”, “Spinal pain disorder.” These search terms were combined using Boolean operators, and the detailed search string is available in Supplementary Table S1. Articles were selected on the basis of relevance to the review’s objectives. The inclusion criteria were as follows: (1) studies addressing AI applications in spine and spinal pain care, with emphasis on relevance to rural or resource-limited settings; (2) published in English as peer-reviewed articles, including cross-sectional, observational, cohort, case–control, randomized controlled trial, and quasi-experimental studies, reviews, and meta-analyses. Meanwhile, both original and secondary evidence were included because original studies enabled assessment of specific AI applications, model development procedures, validation approaches, and reported clinical outcomes. Reviews were included to provide broader evidence synthesis and contextual understanding of the rapidly evolving field.

Studies were excluded if they (1) did not focus on the application of AI in spinal care; (2) were not published as peer-reviewed journal articles (e.g., abstracts, editorials, or commentaries); (3) lacked clear methodology or focused on animal models without relevance to human applications; or (4) were not published in English.

All references were imported into reference management software (EndNote X9, Clarivate Analytics, Philadelphia, PA, USA), and duplicate references were removed. After removing duplicates, we screened titles and abstracts. Two independent reviewers selected relevant studies for full-text assessment using eligibility criteria and resolved discrepancies through discussion. Reflecting the narrative nature of this review synthesis, a PRISMA flowchart was not generated. Two independent reviewers performed this process. Additionally, because this narrative review integrates diverse primary and secondary evidence, a formal systematic review protocol, study-level risk-of-bias assessment, and quantitative meta-analysis were not performed. Nevertheless, the evidence was critically interpreted by considering the methodological characteristics, clinical context, applicability to rural settings, reported outcomes, and limitations of the included studies. Relevant data were extracted into a structured evidence matrix by two independent reviewers, any discrepancies were resolved through discussion.

Due to the presence of significant heterogeneity in the included studies, study populations, AI model types, and outcome measures, a quantitative analysis was not performed, and characteristics and outcomes were presented narratively. A reliability test was performed to evaluate the internal consistency using Cronbach’s alpha. An excellent internal consistency (>0.90) between the two reviewers was observed.

## 3. Rural Inequity in Spine Care

In the US, rural populations face limited access to effective pain management and, in part because of this, a high prevalence of chronic pain [20]. It has been estimated that more than one in five elderly US citizens live in rural settings, a population that faces major health inequities and poorer clinical outcomes [21,22]. Neurosurgery remains one of the most geographically maldistributed surgical specialties in the US. The national density is 1.58 neurosurgeons per 100,000 population, which technically exceeds the WHO benchmark of one per 100,000, yet US neurosurgical care is highly maldistributed [23]. For instance, approximately 9.8% of the US population lives in areas where neurosurgeon density is below 0.5 per 100,000; as a result, more than 32 million Americans reside without local access to essential spine care services [24]. Moreover, 80% of US counties lack neurosurgeons, and 2.3% practice in remote areas, while higher numbers are working in the urban healthcare settings [25]. Lower odds of practicing in rural settings are associated with subspecialization status (OR 0.40, 95% CI 0.36–0.45) and earlier career stage (OR 0.80, 95% CI 0.70–0.91) [26]. Delayed treatment for conditions such as traumatic brain injury and SCI is significantly linked to worse outcomes and disability [27]. In addition to clinical harm, the financial burden of traveling long distances for neurosurgical care disproportionately impacts low-income and minority populations, intensifying regional disparities [24].

Rural populations frequently encounter barriers across the care pathway from early diagnosis to treatment and outcomes compared with urban populations [20]. Higher mortality rates for stroke, trauma, and cancer have also been reported in rural areas [28,29,30]. Rural healthcare is constrained by critical shortages of general and specialist clinicians that delay care and strain local providers [31,32]. Barriers to rural practice include workforce shortages, transportation difficulties, inadequate hospital infrastructure, restricted continuing medical education, and professional isolation [27]. Furthermore, rural hospitals continue to close at alarming rates, compounding local specialist shortages [33]. Socioeconomic disparities compound geographic barriers. Lower household income, higher unemployment, higher uninsured rates, and limited educational attainment are associated with fewer practicing neurosurgeons, indicating that access inequities track with social disadvantage [24]. These disparities contribute to delayed diagnosis, long-term disability, increased healthcare costs, reduced QoL, and poorer functional outcomes.

Against this backdrop, AI tools that extend specialized knowledge and improve efficiency may be a valuable adjunct for rural providers and communities, provided they are validated in those settings. Meanwhile, the relationship between health equity and AI is bidirectional. AI models may help mitigate disparities by extending specialist-informed decision support in remote/rural areas, facilitating earlier recognition of clinically important spinal disorders, supporting remote assessment and follow-up, and even performing more effective spine surgeries [18]. However, challenges remain, including ensuring equitable access to these technologies, managing the ethical implications of AI-driven decisions, and addressing digital literacy gaps [34]. Although AI presents the opportunity for a healthcare revolution, it is essential to address the regulatory, ethical, and safety challenges associated with its integration [35]. AI cannot replace essential healthcare infrastructure or compensate for the absence of surgical facilities, specialist workforce capacity, emergency transportation, rehabilitation services, or reliable systems for follow-up and definitive treatment.

## 4. Current AI Landscape in Spinal Pain Disorders

AI has rapidly expanded across healthcare to support diagnosis, personalize treatment, improve patient outcomes, and assist clinical decision-making (Table 1). In spine care, it has been applied across operative and non-operative contexts, including surgical planning, outcome prediction, and digital self-management of chronic low back pain [36]. The integration of AI is reshaping clinical decision support systems, with the potential to anticipate clinical deterioration and suggest evidence-based next steps [37]. Meanwhile, included studies were retrospective and conducted at single centers, which may limit the representativeness and increase the risk of selection and spectrum bias. In addition, small datasets and limited numbers of outcome events may increase the risk of model overfitting and overly optimistic estimates of performance, particularly when complex ML or DL approaches are used.

### 4.1. Structured Prediction and Phenotyping

A recent scoping review reported that non-operative management of musculoskeletal pain, especially chronic LBP, uses chatbots, mobile applications, exercise-coaching platforms, and ML decision support. Patients often used smartphones and wearable tools to receive personalized exercise prescriptions, patient education, and cognitive behavioral therapies, with intervention durations ranging from two weeks to one year. These interventions showed modest benefits in pain, disability, and quality of life. In operative contexts, AI and augmented reality (AR) applications have been used for patient selection and intraoperative navigation in spinal cord stimulation [36]. AI models can detect key spinal pathologies with high reported performance, including stenosis, fractures, tumors, and infections. AI can also assist with surgical planning through CT synthesis, AR systems, and robotic guidance. When combined with clinical data, these models can support personalized treatment predictions [38]. In a pilot study, an ML algorithm was used for diagnosis of spinal pain disorders in 246 patients, with an accuracy of 72% [39]. With the continuing expansion of clinical data, electronic health records, genomics, and wearable sensors, these technologies are increasingly integrated into spine research to enhance decision-making [40]. Such models can provide more personalized estimates of future clinical trajectories, accounting for the multifactorial nature of spinal pain disorders.

Pain intensity remains one of the most frequently incorporated variables in AI prediction models [41]. Pain intensity is measured using standardized tools such as the visual analogue scale, brief pain inventory, and numeric rating scale, which quantify severity and can be analyzed longitudinally to identify pain trajectories over time [42].

### 4.2. Imaging AI

AI has emerged as an important tool for spinal imaging, providing automated, reproducible, and precise analysis that supports spine surgery [43]. Machine learning (ML), a subtype of AI, is expanding its role in spine surgery, particularly in radiological image analysis, including labeling and localization of anatomical structures and the detection and classification of radiological findings [44]. Advances in AI, particularly deep learning (DL), are crucial for innovation in spine surgery. Several studies report AI applications in spine imaging that improve diagnostic accuracy and support complex procedures with greater precision [45]. An ML classifier (Gaussian Naïve Bayes) achieved 79% accuracy in identifying patients with chronic LBP, with the most discriminative feature being the angular velocity of flexion [46]. ML can also automate interpretation of intraoperative images and provide preoperative guidance for adult spinal deformity [47]. DL models based on convolutional networks extract imaging features that high-performance classifiers then categorize to support diagnosis. These models have been applied to segmentation, detection, diagnosis, and quantitative assessment of spine images [48], and have achieved high accuracy in diagnosing conditions such as scoliosis [49], osteoporosis [50], spinal stenosis [51], and spinal cord injury [52].

**Table 1:** Summary of AI-based tools for prediction, diagnosis, and treatment of spinal pain disorders.

AI-Technique	Sample Size (N)	Study Setting	Validation Method	External Validation	Clinical Domain	Spinal Pain Disorders	Performance Metrics (e.g., Accuracy, AUC, Others)	References
AI/ML	<1000 patients	Systematic review of published studies	No validation model performed	No	Prediction and treatment response	Pain, LBP	Accuracy ranged from 61% to 100%	[53]
ML	82 patients	Multicenter cohort	10-fold cross-validation; expert radiologist as reference standard	No	Spine imaging	Lumbar spinal stenosis	Sensitivity 94%, specificity 98%	[54]
Generative AI (ChatGPT)	31 included studies	Scoping review (studies conducted across healthcare settings)	N/A	No	LBP treatment	LBP (non-specific LBP and specific spinal pathologies)	No pooled performance (accuracy/AUC) reported. GenAI models produced better agreement in clinical guidance. However, errors were reported, including hallucination and low accuracy	[55]
SVM, RF, kNN	62 patients	Publicly available dataset	Internal validation	No	LBP prediction using lumbar imaging	LBP	66–72% accuracy	[56]
DL (Tiramisu NN and CNN)	500 patients	Single-center retrospective MRI study	10-time 10-fold cross-validation	No	Spine imaging	Lumbar intervertebral disc degeneration	86% overall accuracy	[57]
CNN	542 patients	Single-center retrospective MRI study	Expert comparison (Internal validity)	No	Spine MRI	Spinal stenosis	Achieved 89–91% agreement with expert grading	[58]
SVM, ANN, kNN, Naïve bayes, Decision tree	33 patients	Single-center retrospective electromyography study	10-fold cross-validation	No	Clinical decision support	Lumbar disc herniation	Naïve bayes and Decision tree showed high accuracy	[59]
SVM	167 patients	Single center	Internal validity	No	Diagnosis, Prediction	LBP	Accuracy = 78–92%, AUC = 0.78–0.97	[60,61,62]
AI-based clinical decision support system	34 patients	Single-center pilot study	No internal validity	No	Clinical decision support	LBP	AI tool successfully generated diagnosis, prognosis, and treatment recommendation	[63]

Abbreviations: ML: Machine Learning; DL: Deep Learning; SVM: Support Vector Machine; RF: Random Forest; kNN: k-Nearest Neighbors; AUC: Area Under Curve; LBP: Low Back Pain; NN: Neural Network; CNN: Convolutional Neural Network.

**Figure 1:**
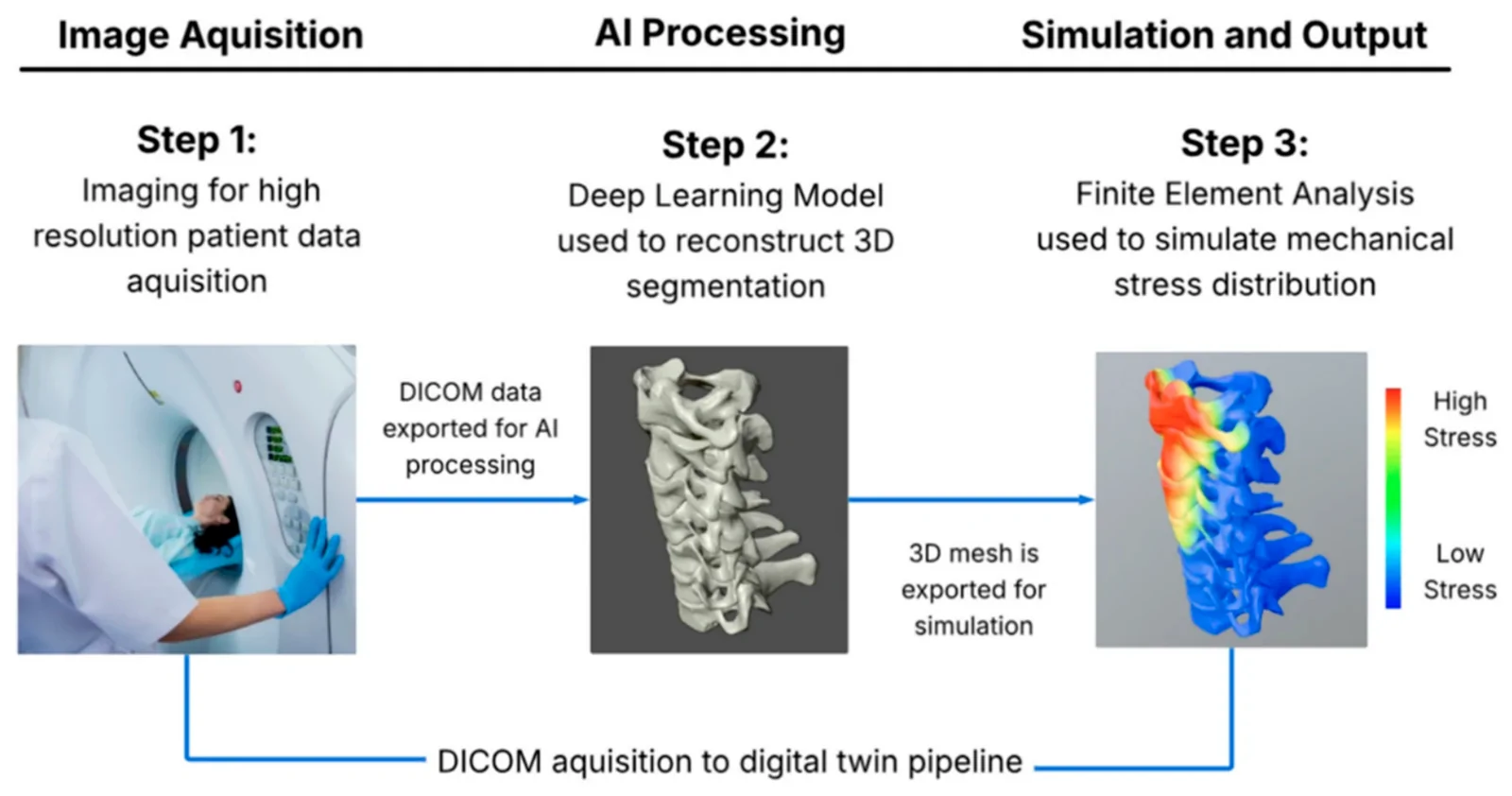
Process of digital-twin creation using deep-learning algorithms, licensed under the terms of Creative Commons Attribution (CC BY) 4.0 [68].

Several studies report DL models for automatic detection of lumbar disc herniation on MRI with accuracy up to 96.7% [64]. A convolutional neural network (CNN) model performed automated categorization of lumbar spinal stenosis severity on MRI, with high accuracy concordant with expert radiologists [65]. In another study, two CNN models one grading sagittal MRI into five clinically relevant grades and one detecting stenosis, both achieved accuracy and recall of 97–98% [66]. Generative AI has been used to construct 3D CT images from 2D X-rays, extending diagnostic capacity while reducing patient exposure to CT radiation [67]. A recent review reported that AI-assisted robotic pedicle-screw placement achieves clinically acceptable accuracy and reduces radiation exposure to staff, although it may increase operative time [45]. Figure 1 illustrates the digital-twin creation process in spine surgery. Although digital twins represent a promising future direction for personalized spine care, their current implementation in rural healthcare settings remains challenging.

Overall, these studies demonstrate the potential of AI to enhance the efficiency and accuracy of spinal-disorder detection on imaging and to reduce variation between clinical interpreters. However, most AI models are trained in tertiary centers using high-quality imaging data, and their diagnostic performance may be influenced when deployed using the low-quality and heterogeneous imaging typical in rural settings. Close collaboration between spine surgeons, rural healthcare providers, and AI researchers will be needed to translate these tools into clinical practice.

### 4.3. NLP and LLM Applications

**Figure 2:**
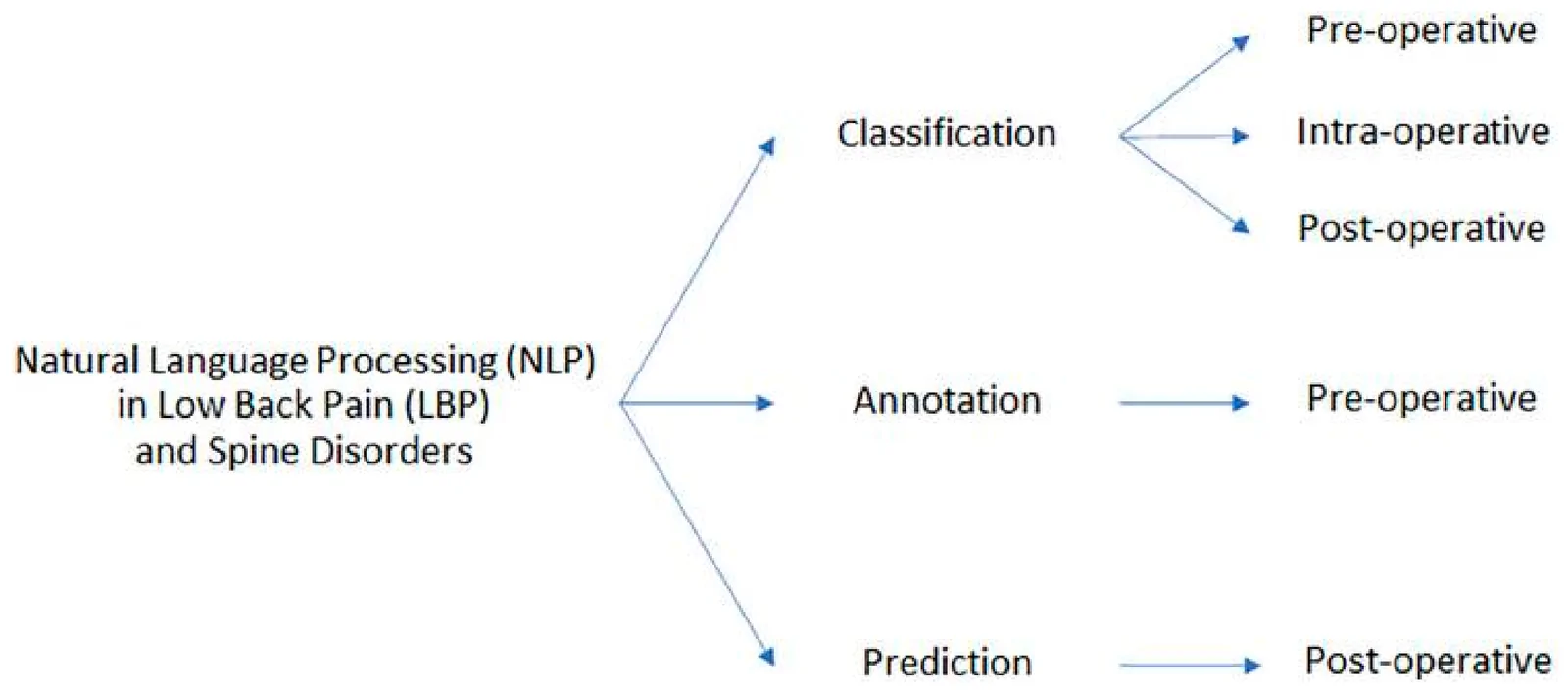
Schematic representation of NLP applications in LBP and other spinal pain disorders, under the terms of Creative Commons Attribution (CC BY) License [69].

Early applications of AI in spinal pain research focused on medical image analysis and predictive modeling; more recent advances involve natural language processing (NLP), a discipline at the intersection of AI, computer science, and linguistics that analyzes human language [69]. Figure 2 summarizes pre-, intra-, and postoperative NLP tasks.

NLP, a branch of ML, can extract information from unstructured text and support predictive modeling. Free-text reports have been analyzed to predict clinical outcomes such as intraoperative vascular injury in anterior lumbar spine surgery [70], and readmission after posterior lumbar fusion [71]. NLP system showed higher sensitivity in identifying standard reporting characteristics for LBP on radiographic imaging than traditional rule-based models [72]. A rule-based NLP system also showed good diagnostic performance (accuracy, precision, recall, F1) in identifying radiological findings such as disc bulge, listhesis, disc extrusion, annular fissure, disc protrusion, Modic type 1 change or endplate edema, Schmorl node, lateral recess stenosis, osteophyte, and stenosis associated with LBP [73]. NLP has also been used for clinical decision-making, postoperative opioid monitoring, patient counseling, and research-registry development [74].

Large language models (LLMs) represent the next evolution of NLP. Examples include ChatGPT, Gemini, and Claude, which generate human-like text and have shown promise in supporting the assessment of various spinal conditions using advanced pattern recognition [75]. By combining NLP and ML, LLMs can extract critical data from unstructured sources such as clinical notes and imaging records, yielding insights that may support clinical decision-making [76], and can streamline workflow by reducing manual data entry [77]. LLMs may help interpret MRI for detection of spinal pain disorders. MRI is a key diagnostic tool that provides detailed visualization of vertebrae, discs, spinal cord, and soft tissue, but interpretation can be time-consuming and limited by interobserver variability [78]. By training multimodal LLMs on large radiographic datasets, these models may facilitate automated detection and localization of spine pathologies such as spinal cord injury, spinal stenosis, herniation, and vertebral fracture [79,80,81]. LLMs can generate personalized spine-surgery education tailored to patient characteristics, and can interpret imaging reports and classify scoliosis severity from textual descriptions [82]. LLMs can draft patient-facing documents, such as LBP evaluation templates and consent forms [83], and support classification, patient counseling, and research [74]. A BERT-derived spinal LLM improved classification of lumbar spine disorders [84], and a retrieval-augmented model (RAG-LLM) produced more accurate, guideline-adherent responses for clinical decision support [85]. However, clinician validation of generated text remains necessary. Overall, these models can interpret extensive medical literature, patient data, and expert knowledge to support diagnosis and planning for complex spinal pathology and may serve as a supplementary tool [86,87]. However, comprehensive research is needed to fully assess LLM performance across spinal disorders.

### 4.4. Remote Monitoring and Digital Rehabilitation

Remote monitoring is an increasingly important application, allowing providers and patients to maintain contact between visits. After spine care, post-treatment monitoring and rehabilitation play a crucial role in preventing complications and recurrence. AI models can analyze diagnostic imaging and clinical data to suggest customized rehabilitation programs based on individual anatomy and injury severity [88]. AI applications in post-treatment monitoring may improve adherence, reduce costs, and improve functional recovery [8].

AI systems, like NXTSTIM EcoAI^TM^, are used for the continuous monitoring of patients, timely interventions, and improved clinical outcomes [89]. Such AI systems can be integrated into remote monitoring technologies, such as mobile applications and virtual reality platforms, expanding high-quality rehabilitation programs to remote and underserved patient populations. These smart platforms used real-time correction features to stimulate direct therapy sessions, significantly reducing the clinical visit time for postoperative patients, such as lumbar discectomy patients [90].

AI-powered wearable braces, motion sensors, and fitness trackers enable monitoring throughout rehabilitation by tracking gait, activity, and range of motion. These algorithms analyze the collected data to detect deviations from expected healing, allowing clinicians to identify complications early and act promptly [91]. AI-powered wearables can also detect posture and monitor spinal alignment in real time, helping patients maintain correct movement patterns and reduce back strain [92]. Remote monitoring can flag early signs of delayed recovery, such as decreased mobility or increased pain, alerting clinicians to intervene before complications develop [93]. Continuous AI monitoring provides a more comprehensive picture of patient status than intermittent clinic visits alone. ML can also detect inconsistent activity and trigger customized reminders, motivational messages, and exercise modifications to enhance rehabilitation adherence [94].

## 5. General Purpose and Clinician-Facing AI Tools

The rapid evolution of AI has produced a diverse ecosystem of tools that differ in their use, access to medical evidence, and role in care. These tools are categorized into general-purpose systems, specialized clinical decision-support systems, and task-specific imaging or predictive models. Distinguishing between them is essential, as these technologies differ significantly in their evidence base, infrastructure requirements, clinical maturity, and potential risks. General-purpose LLMs such as Gemini, ChatGPT, and Claude are designed primarily for text generation and language understanding. Their strengths lie in natural-language understanding, explanation of complex concepts, summarization, information organization, and drafting clinical documentation [95]. A recent study analyzed responses of four LLMs (ChatGPT-4.0, Gemini-1.5 Pro, Claude-3.5 Sonnet, and Llama-3.1) related to 37 spinal-cord-injury questions spanning pathogenesis, diagnostics, clinical features, prognosis, treatment, and risk factors. Response quality was measured with the Ensuring Quality Information for Patients (EQIP) tool and Flesch–Kincaid metrics. Readability across all four models was low (college-level reading required), but comprehensiveness was high. Gemini ranked highest in overall quality, while ChatGPT was most accurate [96]. Another study examined LLMs for calculating the Spinal Instability Neoplastic Score (SINS): LLM-based applications, whether fully automated or clinician-assisted, provided high accuracy and efficiency, reducing scoring time while maintaining consistency, potentially accelerating cancer-care pathways for patients with spinal metastases [97]. These LLMs can be useful for rural clinicians who have limited immediate access to specialist resources. However, LLM integration into clinical practice remains challenging. Challenges include workflow misalignment, as rural areas have lower populations compared to urban areas, leading to different demands, also rural healthcare settings face severe shortage of spine specialists, diagnostic safety, equity and bias, legal and regulatory issues, technical vulnerabilities such as data poisoning, and preservation of patient trust and human connection [98]. These models may produce incorrect or hallucinated outputs, are difficult to interpret (the “black-box” problem), carry biases from training data, and raise complex ethical and regulatory questions [99]. This may be due to limited training in interpreting complex algorithmic outputs and fewer opportunities for immediate technical or specialist consultation. Moreover, translation of AI models into practice requires advances in reliability, privacy protection, operational integration, and governance [100].

By contrast, clinician-facing AI platforms are developed to support evidence-informed practice. Rather than relying on open-ended text generation, many emphasize evidence retrieval, transparent citations, integration of current clinical guidelines, and decision support within the workflow. One example is OpenEvidence, which draws on trusted sources to generate evidence-based recommendations and is rated highly for relevance, clarity, and evidentiary support [101]. OpenEvidence serves as a reliable, fast tool for answering clinical inquiries regarding labs to consider, treatment options, alternative treatments, and updated guidelines. It is also helpful in finding more evidence and developing more appropriate treatment plans [102]. OpenEvidence performed better than ChatGPT-4o, with an acceptable response rate of 100% in OpenEvidence, while it was 92.5% in ChatGPT-4o [103]. Likewise, OpenEvidence also outperformed Gemini 3.1 Pro, GPT-5.2, and Claude Opus 4.6 [104]. Additionally, ChatGPT can be helpful in environments with limited access to trained surgeons or in rural healthcare settings [105].

## 6. A Translational Model from Centers of Excellence to Rural Practice

AI and LLMs can assist with clinical documentation, diagnosis, patient communication, and electronic health records (EHRs). ML algorithms support predictive modeling for chronic-disease pattern recognition and remote patient monitoring (RPM). AI-driven RPM systems have played important roles in managing chronic diseases such as cardiovascular disease, diabetes, and cancer in rural areas, providing continuous monitoring and timely intervention through wearables and mobile applications [106]. A major challenge in translating such innovations into routine spinal pain care is that most AI models were developed and validated in urban settings with large medical centers, whereas rural settings lack such infrastructure [107]. These techniques carry data, computational, and regulatory requirements that directly affect feasibility and adoption in rural areas. Despite advances in AI, deployment faces critical barriers including data quality, patient privacy, inadequate training data, accessibility, and compliance. In rural areas with limited infrastructure, staff shortages, geographic isolation, and longer access times well-validated AI could nonetheless help improve access, diagnosis, and quality of care [108]. Improvements in EHR infrastructure, workforce experience, and computational resources are needed to deploy AI in rural services [19]. Simply transferring an urban-trained model into a rural setting is unlikely to produce optimal performance [107]. Successful translation requires adaptation to frontline realities: rural clinicians often work with incomplete diagnostic information, limited imaging, and fewer specialist services. AI systems intended for rural use must be efficient, interoperable with existing EHRs, and critically able to function with incomplete data. In prior work by the authors, a ML decision-support tool for rural spine care was developed and presented as a poster titled “Leveling the playing field in rural health care by implementing an artificial intelligent machine learning algorithm to accurately diagnose spinal pain conditions” at the Dartmouth Rural Health Symposium (12–13 May 2026). In the target region, many patients live in rural areas where the lack of local specialists and diagnostic tools increases the risk of misdiagnosis and delayed treatment for complex spinal conditions. The tool was trained on 250 patients across four spinal diagnoses including lumbar radiculopathy, lumbar spondylosis without myelopathy, post-laminectomy syndrome, and sacroiliitis, using 80 clinical variables including pain score, pain duration, symptom location, and prior interventions (physical therapy, injections, surgery). The model is intended to support rural physician judgment and guide therapeutic decisions, with ongoing work focused on optimizing predictive variables and expanding the dataset for clinical validation [109]. The authors presented the tool in its preliminary form at the symposium. The underlying evidence is based on an initial pilot dataset, and the tool has not yet undergone peer review, independent external validation, or prospective clinical evaluation. Therefore, it should not be interpreted as an established or validated clinical-support system, should not replace physician judgment, and may improve diagnostic consistency for complex spinal conditions in underserved communities. Importantly, this is an early-stage pilot with several limitations, including a small sample size, limited data on some variables, and most importantly, the absence of external validation. The interpretation of pilot results should be made with appropriate caution until full peer-reviewed data becomes available.

Continuous learning should form the final component of the translational framework. Such systems should undergo performance monitoring, iterative refinement, and periodic recalibration as new local data become available. Establishing bidirectional feedback between academic centers and rural providers would allow the model to evolve with the population it serves.

### 6.1. Stages of Development of AI-Based Models

#### 6.1.1. Building a High Quality Base of Knowledge

**Figure 3:**
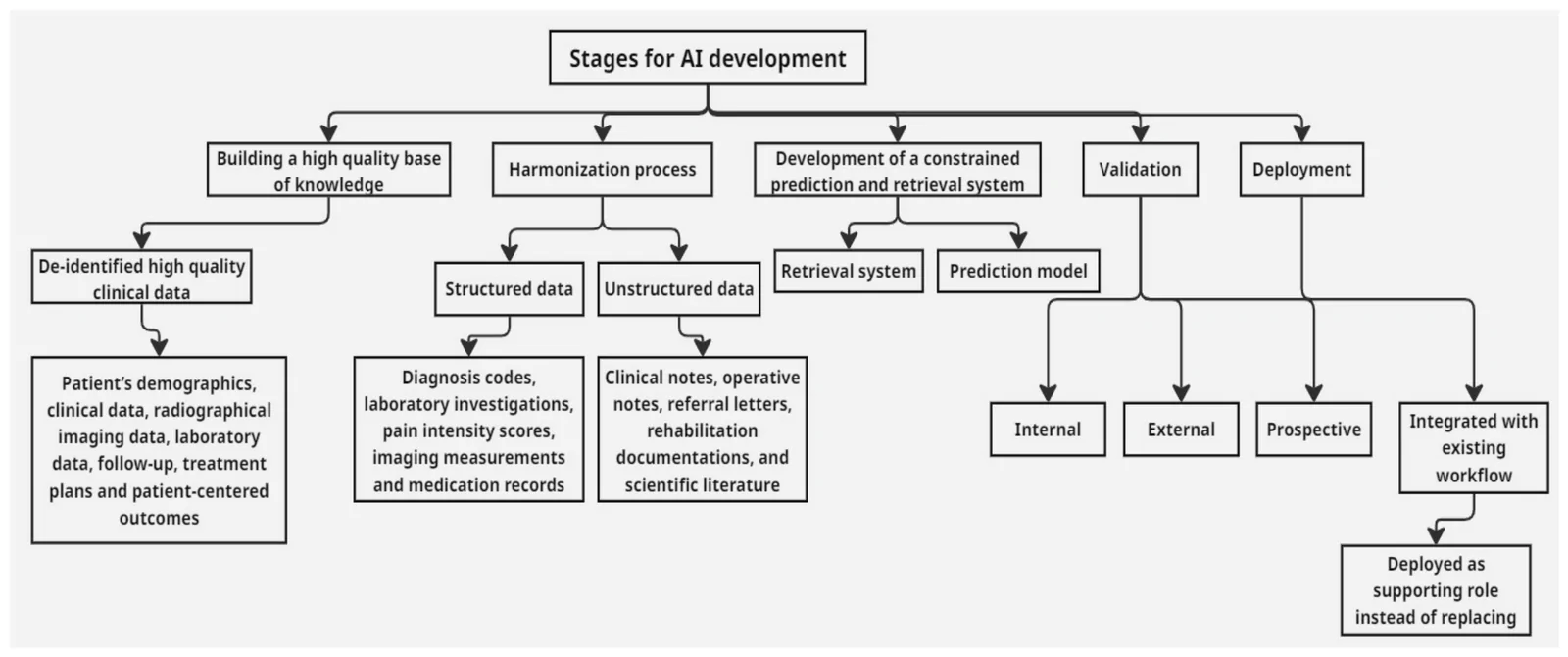
Developmental steps for a translational AI model for rural healthcare settings.

The proposed model begins with collection and extraction of de-identified, high-quality clinical data from centers of excellence [110]. Rather than relying on data from a single center, aggregating data from multiple centers allows the AI system to learn from a large, diverse population spanning a wide range of spinal pain disorders, from uncomplicated mechanical back pain to more complex conditions such as traumatic injury, inflammatory disease, spinal tumors, and infection. Centers of excellence generate standardized clinical data through multidisciplinary care involving surgeons, neurologists, pain specialists, physiatrists, radiologists, and physiotherapists [111,112,113,114]. Data should include demographics, clinical variables, imaging, laboratory results, follow-up, treatment plans, and patient-centered outcomes [115]. Meanwhile, before developing the model, participating sites should establish common data definitions, assess completeness and quality, document data provenance, and define clear procedures for securing data sharing or privacy-preserving collaborative learning. Additionally, including rural sites at this stage is also very important to avoid developing models based exclusively on urban or tertiary healthcare populations. Collectively, such data enables the model to learn not only diagnosis but also predictors of clinical outcome (Figure 3).

#### 6.1.2. Harmonization Process

In the second step (Figure 3), structured data (diagnosis codes, laboratory results, pain scores, imaging measurements, medication records) [116] and unstructured data (clinical notes, operative notes, referral letters, rehabilitation documentation, and literature) are harmonized into standardized datasets suitable for AI training [44]. Harmonization enables models to learn from the complexity of routine clinical practice rather than relying solely on isolated numerical variables. During harmonization, structured variables, relevant unstructured clinical information, and imaging data should be mapped to common definitions and standardized terminology to make information usable and comparable across different systems. This approach is helpful in increasing the statistical power of a dataset to solve issues that cannot be addressed using data from a single study [117]. Likewise, missingness should be characterized rather than automatically treated as a random and AI models should be tested under realistic conditions of incomplete documentation. For the management of missingness, data-tailored imputation strategies, such as last observation carried forward for repeated measures evolving to plateau over time, and characterization of the missingness pattern [118].

#### 6.1.3. Development of a Constrained Prediction and Retrieval System

The third step develops a constrained prediction-and-retrieval system. This improves accuracy and reduces hallucination by retrieving external knowledge and grounding the model’s responses in it [119]. Prediction models estimate probabilities of clinical outcomes such as diagnosis, prognosis, and personalized treatment [120]. Predictive models may use appropriate statistical or ML approaches and should be compared with conventional clinical baselines. This stage should also incorporate separate training and validation datasets, subgroup analysis, calibration assessment, and estimation of uncertainty. Retrieval complements prediction by identifying similar patient cases and presenting supporting evidence from comparable scenarios [121]. Retrieval-based systems should use traceable evidence sources, allowing clinicians to verify the generated information. Together, these create an evidence-based decision-support environment that lets clinicians understand the basis for each recommendation (Figure 3). Meanwhile, when available information is insufficient, AI models should flag uncertainty rather than generating potentially misleading outcomes.

#### 6.1.4. Validation

The next step is validation. Data, development, and interaction biases can arise from training data, feature engineering, algorithmic and clinical factors, and reporting or temporal effects [122]. Both internal and external validation should be performed to prevent overconfident predictions, minimize hallucinated recommendations, and ensure alignment with evidence-based guidelines. The most important element of the framework is prospective validation within rural settings (Figure 3), because models developed in urban or tertiary settings cannot be assumed to perform well in isolated rural environments [123]. During prospective validation, measures such as calibration, discrimination, decision curve analysis, the Brier score, sensitivity, specificity, and predictive values should be considered [124,125]. Sample size requirements should be determined prospectively, as no standard tool exists for clinical validation studies of AI models; however, some open-source methods for sample size analysis can be used [126]. Meanwhile, a major limitation of AI models is the uncertain external validity [36]. A previously published review reported that most AI models used in spine were trained on single-center, small-sample datasets and had limited validation [10]. Current models are also heavily skewed toward spine imaging and lack key clinical, patient-specific, and outcomes validation. Furthermore, datasets used to develop these models are limited by poor external validation [127]. A meta-analysis also identified no externally validated models suitable for clinical use regarding traumatic fractures of the cervical spine and traumatic or osteoporotic fractures of the thoracolumbar spine in non-emergency settings [128]. External validation is important because it uses datasets from multiple hospitals, covering diverse types of scanners, patient groups, and settings, to ensure the model works well in varied clinical settings [129]. This is well supported by the findings of a meta-analysis, which concluded that internally validated AI models in radiology had sensitivity > 85% and specificity > 68%, while in external validation it tends to underperform [130]. Therefore, high performance during model development or internal validation should not be interpreted as evidence of clinical generalizability to rural-care settings. Mandatory external validation on diverse cohorts and cautious clinical integration are recommended [130].

#### 6.1.5. Deployment as a Clinician-Facing Decision Support Layer

Following internal, external, and prospective validation, the model can be integrated into existing clinical workflows in rural settings as a clinician-facing decision-support layer. The model should function unobtrusively during routine care and provide support during decision-making rather than replacing clinician expertise (Figure 3). Furthermore, deployment requires implementation planning rather than simple installation of the model. Such planning includes workflow mapping, usability testing, interoperability with existing systems, clinician training, low-bandwidth functionality, technical support, and clear human oversight procedures that should be established before final deployment. Meanwhile, AI outputs may include:

- Ranked differential diagnoses or early detection strategies based on the patient’s clinical features;
- Identification of red-flag conditions requiring urgent evaluation;
- Evidence-based management recommendations after diagnosis;
- Management strategies aligning with currently used clinical guidelines.
- Referral triggers.
- Recommendations for follow-up intervals and monitoring of care of patients.

For AI clinical decision support systems to be safely translated into rural spine care, the implemented framework should extend beyond the model development and address the full pathway from technical evaluation to real-world deployment. Model performance should not be assessed solely based on overall accuracy. Although explainability techniques are widely applied, they often lack clinical validation, and their contribution to trustworthiness is limited when considered in isolation [131]. Prospective validation in real-world healthcare settings is required for the assessment of the impact of AI models on their diagnostic performance [132]. In addition, appropriate human oversight and explainability should be considered and incorporated into the development of AI models [133]. AI recommendations should support rather than replace clinical judgment, as they can strongly influence diagnostic decisions, and when correct, they improve accuracy, but when incorrect, they reduce accuracy, indicating overreliance on the system and posing a significant safety risk [134]. Therefore, AI models should incorporate mechanisms to identify insufficient or out-of-distribution inputs and, when appropriate, flag predictions for clinician review of automated recommendations. Another important requirement for efficient operation of AI models is interoperability, which can help implement AI models in more heterogeneous rural healthcare settings, these models should integrate with existing electronic health records and patient-reported outcomes using standardized, and transferable data structures [135]. Collectively, these measures can help ensure AI systems demonstrate not only strong predictive performance but also transparency, reliability, safety, and practical applicability in real-world rural spine-care settings.

## 7. Safety, Bias, Privacy, and Regulations

Successful deployment of AI decision support depends on continuous access to large, accurate, standardized data. Although AI models have shown improvements in diagnosis and evidence-based management, they carry risks that are important in rural settings, where foundational health data are limited, fragmented, and inconsistently captured across community institutions. This data scarcity, together with limited EHR interoperability, prevents rural clinics from sharing diagnostic information and building longitudinal patient profiles [136]. Rural populations may also be underrepresented in training datasets, particularly older adults, potentially resulting in biased predictions, poorer calibration, or reduced diagnostic accuracy when models are transferred from urban healthcare settings. For instance, AI triage systems trained predominantly on specialist center data, when applied to patients with different demographics, characteristics, or patterns of healthcare access, it can result in inappropriate outcomes [137]. Due to this limited data availability is a central concern for robust model development, validation, and deployment [19]. Meanwhile, realizing the full potential of AI models in rural settings remains challenging in socioeconomic and infrastructural terms, and requires active collaboration [138]. Therefore, strengthening rural representation requires intentional inclusion of these settings, standardized prospective data collection, subgroup-specific performance assessment, and documentation of data provenance. When direct sharing of data from rural healthcare settings is limited by privacy or institutional constraints, then privacy-preserving approaches, such as federated learning should be applied, in which models are trained on datasets without transferring identifiable patient data [139]. Additionally, patient’s data can be secured by providing role-based access controls, encryption of data transmission or storage, multifactor authentication, regular software updates, audit trails, secure backup procedures, and incident-response plans that are feasible for resource-constrained or rural healthcare settings.

One of the most widely reported concerns is hallucination, particularly in LLM-based systems such as ChatGPT, which can produce incorrect information [140]. Relatedly, premature closure is a risk: clinicians may accept a confident AI output without adequately considering alternative explanations [141]. Meanwhile, AI models should communicate uncertainty, identify inputs outside their validated range, allow clinician override, and include clear escalation pathways for uncertain or high-risk cases.

Data quality is an equity issue, not merely a technical one. Older, socioeconomically vulnerable patients with a higher burden of complex conditions are systematically underrepresented in mainstream registries; models trained on such data may be less accurate and biased when deployed in rural settings [136]. Algorithmic bias is a further concern: where error margins are thin, and exclusion is costly, biased models can widen existing health gaps rather than close them [142].

Privacy and data governance are other critical priorities in the implementation of AI models, as they can analyze the most complex datasets, including patient’s personal information via electronic health records, genomic profiles, and radiological images [115]. Wearable devices and mobile health applications empower patients to monitor their health, and such devices can collect most sensitive health data, leaving patient’s personal data vulnerable to data breaches and identity theft, to counter such risks, there is intensified need for robust cybersecurity [143]. For this purpose, regulatory requirements should also be considered before implementing AI models, including oversight of AI-enabled medical devices by relevant authorities such as the FDA or other specific governing bodies. Finally, the need for a large and representative dataset must be balanced against privacy concerns, such as secure distributed learning, collaborative data governance agreements, privacy-enhancing approaches, and data minimization may enable broader model development while helpful in reducing unnecessary exposure of sensitive patient information.

Furthermore, technical risks primarily associated with the robustness and reliability of AI models and individual sources of risk can be evaluated and analyzed for criticality to define suitable mitigation strategies for technical risk management at an early stage, leading to a robust and reliable system [144]. Rural healthcare settings may generate a different set of information that can be misleading, and this is also considered a major limitation in the USA, as no studies have been found to validate or train in rural settings [19]. In addition, clinical risks may arise when AI-generated outputs influence patient assessment, diagnosis, triage, and treatment decisions, while misleading recommendations may contribute to diagnostic errors and inappropriate triage [137].

Overall, human oversight remains the cornerstone of safe implementation of AI. It should be used as a complementary, supportive manner instead of replacing clinical expertise.

## 8. Future Directions

This review shows that AI extends well beyond image interpretation and prognostic prediction to encompass clinical decision support and personalized treatment planning. These capabilities are especially relevant to rural settings, where limited diagnostic resources, specialist shortages, and fragmented care pathways impede timely access to evidence-based care. Future progress will depend on the following priorities:

- The immediate future objective for the implementation of AI models should not be to replace specialists or clinical experts. AI should be viewed as a supportive role that strengthens clinical expertise rather than replacing them.
- Many published AI models demonstrated impressive performance under retrospective conditions, without rigorous evaluation within clinical environments, including rural healthcare settings. Future studies should focus on prospective rural validation, and these validations should examine not only discrimination and calibration but also workflow integration, clinician acceptance, fairness, safety, and notably its performance in incomplete or fragmented electronic health records.
- AI models should also be trained and validated in multicenter settings, including rural healthcare settings, diverse imaging equipment, and variable data quality.
- Sample size should be determined prospectively based on intended clinical outcomes, model complexity, event frequency, and desired precision rather than applying a universal threshold.
- Future studies should develop multimodal AI models that integrate multiple complementary sources of information associated with clinical data rather than relying on single isolated datasets. Such multimodal models should integrate electronic health records, laboratory investigations, clinical notes, telehealth assessments, wearable sensor data and patient-reported outcomes.
- Another most important future research priority should be the implementation of AI models, which seek to assess whether these models produce measurable improvements in the care of patients. It should not be limited to accuracy, other important parameters, like appropriateness of specialist referrals, adherence to evidence-based guidelines, reduction in unnecessary imaging, earlier detection of red-flag conditions, satisfaction of patients, improvement in pain intensity, functional recovery, QoL, and time to definitive treatment.
- Future studies should also examine how AI models can be adopted in rural workflows, including limited specialist availability and fragmented referral pathways.
- Future studies should also compare outcomes of AI models in urban and rural healthcare settings.
- Technically, future studies should also focus on performance of the models when clinical data are incomplete, low-quality images, or inconsistent documentation.
- Economic evaluation of implemented AI model should also be done.
- Future studies should incorporate transparent and explainable decision-making process, regular model recalibration, robust audit trails, and strong protection of the privacy of the patients.
- Regarding policy and implementation, research should address governance, accountability, regulatory requirements, sustainable financing, digital infrastructure, and cybersecurity.

## 9. Conclusions

AI has emerged as a promising tool for strengthening clinical decision support in spinal pain disorders, particularly in rural settings. AI has the potential to extend clinician expertise with evidence-based, timely, and patient-centered recommendations, but it cannot replace clinical expertise. Moreover, the evidence reviewed indicates a significant gap between promising technical performance and demonstrated clinical value in rural practice. Models have been developed using retrospective, single-center, and urban healthcare datasets. Therefore, multimodal AI models that are representative of rural settings should be developed and validated prospectively. The ultimate success of these models will depend not on accuracy alone but also on parameters such as referral pathways, functional recovery, pain outcomes, and patient satisfaction. Researchers and technology developers should collaborate and include rural stakeholders to achieve better and more reliable outcomes.

## Data Availability

No new data were created or analyzed in this study. Data sharing is not applicable to this article.

## Supplementary Materials

The following supporting information can be downloaded at: https://www.mdpi.com/article/10.3390/healthcare14182935/s1, Table S1: Literature search across databases.
