# Supplementary material for: Artificial Intelligence for Clinical Decision Support in Rural Spine Care: A Narrative Review
Source: Healthcare (Basel). 2026 Sep 10;14(18):2935. doi: 10.3390/healthcare14182935 (PMC13606791; doi:10.3390/healthcare14182935)
Supplement: Supplementary file 1 [file healthcare-14-02935-s001.zip › healthcare-4510815-supplementary.pdf]

Supplementary Table S1. Literature search across databases.

Databases	Search terms (Free text and MeSH Terms)
PubMed	(("Artificial intelligence"[MeSH Terms] OR "AI tools"[All Fields] OR "machine learning"[MeSH Terms] OR "deep learning"[MeSH Terms] OR "large language models"[MeSH Terms] OR "natural language processing"[MeSH Terms] OR "neural networks"[All Fields] OR "decision support systems"[All Fields] OR "clinical decision support"[All Fields] OR "diagnostic algorithms"[All Fields] OR "digital diagnostics"[All Fields]) AND ("Spine care"[All Fields] OR "spinal cord injuries"[MeSH Terms] OR "spinal pain disorder"[All Fields] OR "spinal disorders"[All Fields] OR "spinal trauma"[All Fields] OR "lumbar spinal stenosis"[All Fields] OR "spinal infections"[All Fields]))
Scopus	TITLE-ABS-KEY (("Artificial intelligence" OR "AI tools" OR "machine learning" OR "deep learning" OR "large language models" OR "natural language processing" OR "neural networks" OR "decision support systems" OR "clinical decision support" OR "diagnostic algorithms" OR "digital diagnostics") AND ("Spine care" OR "spinal cord injuries" OR "spinal pain disorder" OR "spinal disorders" OR "spinal trauma" OR "lumbar spinal stenosis" OR "spinal infections"))
Web of Science	(“Artificial intelligence” OR “AI tools” OR “machine learning” OR “deep learning” OR “large language models” OR “natural language processing” OR “neural networks” OR “decision support systems” OR “clinical decision support” OR “diagnostic algorithms” OR “digital diagnostics”) AND (“Spine care” OR “spinal cord injuries” OR “spinal pain disorder” OR “spinal disorders” OR “spinal trauma” OR “lumbar spinal stenosis” OR “spinal infections”) (Topic)
Cochrane Library	([mh “Artificial intelligence”] OR “AI tools” OR [mh “machine learning”] OR [mh “deep learning”] OR [mh “large language models”] OR [mh “natural language processing”] OR “neural networks” OR “decision support systems” OR “clinical decision support” OR “diagnostic algorithms” OR “digital diagnostics”) AND (“Spine care” OR [mh “spinal cord injuries”] OR “spinal pain disorder” OR “spinal disorders” OR “spinal trauma” OR “lumbar spinal stenosis” OR “spinal infections”)
ScienceDirect	Title, abstract, keywords: (“Artificial intelligence” OR “AI tools” OR “machine learning” OR “deep learning” OR “large language models” OR “decision support systems”) AND (“Spine care” OR “spinal cord injuries” OR “spinal pain disorder”)
Google Scholar	(“Artificial intelligence” OR “AI tools” OR “machine learning” OR “deep learning” OR “large language models” OR “decision support systems”) AND (“Spine care” OR “spinal cord injuries” OR “spinal pain disorder”)
